# L-Asparaginase Isolated from *Phaseolus vulgaris* Seeds Exhibited Potent Anti-Acute Lymphoblastic Leukemia Effects In-Vitro and Low Immunogenic Properties In-Vivo

**DOI:** 10.3390/ijerph13101008

**Published:** 2016-10-14

**Authors:** Saleh A. Mohamed, Mohamed F. Elshal, Taha A. Kumosani, Alia M. Aldahlawi, Tasneem A. Basbrain, Fauziah A. Alshehri, Hani Choudhry

**Affiliations:** 1 Biochemistry Department, Faculty of Science, King Abdulaziz University, Jeddah 21589, Saudi Arabia; melshal2002@yahoo.com (M.F.E.); t.kumosani@yahoo.com (T.A.K.); hchoudhry@kau.edu.sa (H.C.); 2 Biology Department, Faculty of Science, King Abdulaziz University, Jeddah 21589, Saudi Arabia; aaldahlawi@kau.edu.sa (A.M.A.); dr.sema2007b@yahoo.com (T.A.B.); Whitemusk_at@hotmail.com (F.A.A.)

**Keywords:** asparaginase, acute lymphoplastic leukemia, allergy, Immunogenicity, proliferation, apoptosis, cytotoxicity

## Abstract

*Escherichia coli*-derived L-asparaginases have been used in the treatment of acute lymphoblastic leukemia (ALL), however, clinical hypersensitivity reactions and silent inactivation due to antibodies against *E. coli*-asparaginase, lead to inactivation of these preparations in most cases.Therefore, this study was aimed to investigate the cytotoxicity and antitumor effects ofa novel L-asparaginaseenzyme, isolated from *Phaseolus vulgaris* seeds (P-Asp) on the ALL cell line (Jurkat). The immunogenicity of the enzyme was also evaluated in-vivo and results were compared to commercially available enzymes of microbial sources. The data demonstrated that P-Asp has an enhanced anti-proliferative effect on ALL cells as detected by the WST-8 cell viability assay kit. Cells treated with P-Asp also exhibited a higher degree of early apoptosis compared with asparaginase from *Escherichia coli* (L-Asp) or its pegylated form Pegasparagas (PEG-ASP) that induced higher rates of late apoptosis and necrosis as detected by an Annexin V/Propidium iodide binding assay. In-vivo experiments indicated that mice treated with P-Asp had less distinct allergenic responses than other bacterial enzyme preparations as indicated by lower serum concentrations of IgG, IgE, IgM and mMCP-1 compared with other treated groups. In conclusion, P-Asp can be considered as a promising candidate for use in the treatment of ALL.

## 1. Introduction

Acute lymphoblastic leukaemia (ALL) is the most common form of pediatric cancer and it occurs approximately in seven or eight out of 10 children with leukaemia [1]. It originates either from the T- or B-cell lineage, and it is characterized by heterogeneity in numerical and structural chromosomal abnormalities, immunophenotypes, and response to treatment [2]. Several modalities have been proposed for the management of acute leukemia including steroids [3], radiotherapy [4], blood or marrow-derived stem cell transplantation [5] etc., however, the main treatment for childhood ALL is chemotherapy [6].

The chemotherapeutic enzyme L-asparaginase (L-asparagine amidohydrolase E.C.3.5.1.1) is considered a principal component in treatment protocols for childhood ALL that is used for remission induction and intensification treatment in all pediatric and in the majority of adult protocols [7].

The use of L-asparaginase in the treatment of ALL can be traced back to the early studies of Broom et al. who found that the anti-leukemic effect of L-asparaginase is attributed to its ability to hydrolyze L-asparagine, which is required for tumor cell survival [8]. Leukemic cells are unable to synthesize asparagine due to a lack of asparagine synthetase and are dependent on an exogenous source of asparagine for survival [9]. Rapid depletion of asparagine as a result of treatment with L-asparaginase leads to cell cycle arrest and cell death by apoptosis [10]. Meanwhile, healthy cells may survive as they are able to synthesize asparagine de novo with the aid of the enzyme L-asparagine synthetase.

Though asparaginase enzyme is widely distributed in animals, plants and microorganisms [11], only L-asparaginases of certain origins possess antineoplastic activity and among the microbial sources, the most commercially notable ones are *Escherichia coli* and *Erwinia* [12]. A third commercially available preparation, PEG-L-asparaginase (Generic name Pegasparaginase), is a chemically modified form of the enzyme, in which native *E. coli* L-asparaginase has been covalently conjugated to polyethylene glycol. PEG-L-asparaginase was approved in 1994 for use in combination chemotherapy for the treatment of patients with ALL who are hypersensitive to the native form of *E. coli* L-asparaginase [13,14].

All commercially available asparaginase preparations were reported to cause an allergic reaction and significant side effects [15], except in the case of pegylated L-asparaginase where an allergic reaction is less observed [16]. However, allergic reactions to this drug are relatively more common and can be serious especially in patients that have received this treatment at an earlier time [17]. Major side effects of L-asparaginase are anaphylaxis, pancreatitis, diabetes, and coagulation abnormalities that may lead to intracranial thrombosis or a hemorrhage [15]. Therefore, new agents that could induce apoptosis of L-asparagine-dependent tumor cells would be of major interest.

We succeeded in isolating L-asparaginase with high specific activity and with no glutaminase activity from the seeds of *Phaseolus vulgaris*, an edible pea bean, instead of microbial sources [18]. The aim of this study was to investigate the ability of L-asparaginase purified from *Phaseolus vulgaris* to induce anti-proliferative, apoptotic cell death to the ALL Jurkat cell line. The immunogenicity of the enzyme was also evaluated in-vivo and these results were compared with those of commercially available bacterial preparations of L-asparaginase.

## 2. Materials and Methods

### 2.1. Enzymes and Reagents

Our enzyme was extracted from *Phaseolus vulgaris* seeds (P-Asp) as we previously described [18]. We found that P-Asp has a specific activity of 850 International Units per milligram of protein (IU/mg protein). Commercially available lyophilized native or *Escherichia coli* (L-Asp) preparation (Elspar, 10,000 IU per vial) was purchased from MERCK (MERCK & Co. Inc., West Point, PA, USA), and pegylated L-asparaginase (PEG-Asp) preparation (Oncaspar, 3750 IU per vial) was purchased from Sigma-Tau Pharmaceuticals (Sigma-Tau Pharmaceuticals, Gaithersburg, MD, USA). The specific activity of L-Asp and PEG-Asp are 225 and 85 IU/mg protein, respectively [19].

Enzyme preparations were diluted in medium (RPMI-1640) or phosphate-buffer saline (PBS) immediately before each experiment to the desired final concentrations. Aluminium hydroxide (Alum), L-glutamine, Acetic acid, Tween-20, NaHCO_3_, Na_2_CO_3_, Hank’s buffered salt solution (HBSS), Propidium Iodide (PI), an Annexin V-FITC apoptosis detection kit and a WST-8 Cell Counting Kit-8 were purchased from Sigma (Sigma Aldrich, Saint-Louis, MO, USA). RPMI-1640, Trypsin/EDTA, Phosphate buffered saline (PBS), Penicillin G and Streptomycin antibiotics were purchased from GIBCO, Invitrogen Corp. (Grand Island, NY, USA). Mice MCP-1, IgE, IgG and IgM ELISA quantitation kits were purchased from eBiosciences (Affymetrix, San Diego, CA, USA).

### 2.2. Cell Line and Culture Condition

Acute human T cell leukaemia cells (Jurkat clone E6-1) were obtained from the American Type Culture Collection (Rockville, MD, USA). Cells were cultured in RPMI-1640, 10% heat-inactivated fetal bovine serum, 100 U/mL penicillin, 100 μg/mL streptomycin and 2 mM L-glutamine. Cells were incubated at 37 °C and 5% CO_2_.

### 2.3. Cell Viability Assay

Cell viability was determined by the WST-8 (2-(2-methoxy-4-ni-trophenyl)-3-(4-nitrophenyl) -5-(2,4-disulfophenyl)-2H tetrazolium, monosodium salt) assay which is reduced by dehydrogenases in cells to give a water-soluble orange coloured product (formazan) [20]. Briefly, 50 × 10^3^ cells were incubated in 96-well microtiter cell culture plates, in the absence (control cells) or in the presence of tested enzymes, in a final volume of 100 μL. After 24 h incubation, 10 μM of WST-8 was added to each well for an additional 4 h. The optical density of the WST-8 orange colour was measured on a multiwell plate reader (BioTek ELx808, BioTek Instruments Inc., Winooski, VT, USA) using filter 450 nm. Cell viability was expressed as a percentage of control. Data are shown as the mean ± standard deviation of triplicate cultures.

### 2.4. DNA Cell Cycle Assay

Cell cycle experiments parallel cultures were prepared in the same way as cell viability experiments so that samples were harvested at 24 h after supplementation with the different enzyme preparations at a concentration of 1 × IC_50_. Cell cycle distribution and apoptosis were determined as we previously described [21]. In brief, 500 µL of nuclear staining solution (containing 50 µg/mL of propidium iodide, 0.1 mg/mL of RNase A, 0.1% w/v Triton X-100 and 1 mg/mL of sodium citrate) were added to 10^6^ cells. The cells were analyzed after incubation in the dark at 20 °C for 30 min. Total DNA content per cell was determined by analysis of fluorescence at 488 nm by using a FACSCalibur flow cytometer using CellQuest Pro software (Pro Software, BD Bioscience, San Jose, CA, USA). A minimum of 10 thousand cells per sample were acquired to ensure adequate data. Fluorescence of the PI was collected in the FL2 channel, equipped with a 585/42-nm band pass filter. Doublets were excluded from analyses by gating on FL2-W/FL2-A primary plots before a histogram analysis of DNA content (analyzed as FL2-A). Data were analyzed with using ModFit software (ModFit software, Verity Software House Inc., Topsham, ME, USA).

### 2.5. Annexin V Apoptosis Detection Assay

Flow cytometry was used to determine various types of cell death including early and late stage apoptosis as well as necrosis by quantifying cell surface annexin V-FITC (Sigma, Saint-Louis, MO, USA) staining as we previously described [22]. Briefly, cells were seeded in 96-well plates at a density of 5 × 10^4^ cells/mL per well and incubated for 24 h. Cells were treated with the different enzyme preparations at a concentration of 1 × IC_50_, obtained from the WST-8 assay. Afterward, cells were harvested by centrifugation (HeraeusMultifuge X3R, Thermo Fisher Scientific, Villebon-sur-Yvette, France) at 1800 rpm for 5 min then the supernatant was removed. The cells were washed with 200 µL of 1x binding buffer. After removal of the supernatant, 95 µL of binding buffer and 5 µL Annexin V-FITC were added and the mixture was incubated in the dark for 15 min at room temperature. Then 95 µL of binding buffer and 5 μL of propidium iodide (final concentration of 2 μg/mL per cell sample) were added to cells and the mixture was incubated for 15 min in dark at the room temperature. Cells were re-suspended in 200 μL of binding buffer. The stained cells were analyzed immediately by a flow cytometer (NAVIOS Software, Beckman Coulter Inc., Brea, CA, USA) using Navios acquisition Software (Beckman Coulter Inc., Brea, CA, USA). Data were analyzed using Summit Software v4.3 Build 2445 program (Summit Software, DakoCytomation Inc., Carpinteria, CA, USA).

### 2.6. Asparaginase Sensitization Protocol

Thirty female BALB/c mice (albino, laboratory-bred strain) weighing 17–20 gm were divided into five groups (six per cage). The first group is negative control (non-sensitized) that received normal saline, while the rest of animals were sensitized using the adjuvant aluminum hydroxide on days 0 and 14 of treatment. Each of mice groups from the third to the fifth group received adjuvant plus 10 μg of asparaginase preparations (2.25–2.5 IU per mouse) as previously described [23]. Induction of asparaginase allergies in sensitized mice was performed by challenging with a 100 μg i.v. dose of asparaginase preparations on day 24 of treatment. Pre-challenge plasma samples for determining anti-asparaginase antibody levels were collected on day 14 of treatment by retro-orbital puncture, and post-challenge samples were collected by cardiac puncture at the end of the experiment (day 28). Animals were fed *ad libitum* (food available at all times) and maintained under a 12:12-h light/dark photoperiod at 22 °C for 4 weeks. All animals were treated using institutional animal care and use committee (IACUC) approved protocols in accordance with National Institutes of Health guidelines [24]. King Abdulaziz University abides by Royal Decree No. M/59, 24 August 2010 entitled “Research Ethics for Handling of Living Animals”.

Serum samples for an ELISA assay were obtained by centrifugation at 400× *g* and 4 °C for 10 min and stored at −80 °C until analysis. The measuring of antibody levels and mouse mast cell protease 1 (mMCP-1) levels was performed as previously described [23]. Briefly, 100 μL of standards, blank or diluted serum samples were added into wells of 96-well plates precoated with each specific antigen. After incubation for 1 h, 100 μL of enzyme-antibody conjugate was added into each well. Subsequently, 100 μL of TMB (3,3′, 5,5′-Tetramethylbenzidine) substrate solution was added into each well, and incubated at room temperature for 10 min. Finally, 100 μL of stop solution was added into each well, and the absorbance (450 nm) was measured using an ELISA Reader (BioTek ELx800, BioTekInstruments Inc., Winooski, VT, USA).

### 2.7. Statistical Analysis

Results of cytotoxicity, cell cycle and annexin-v/PI assays were presented as means ± SD (standard deviation). Comparisons between multiple groups of animals were carried out using one-way ANOVA followed by a Bonferroni post hoc test. Statistically significant differences between groups were defined as *p*-value < 0.05. The data were analyzed using the SPSS software (SPSS Software, Chicago, IL, USA).

## 3. Results and Discussion

For more than four decades, L-asparaginase preparations derived either from *Escherichia coli* or *Erwinia chrysanthemi*, have been used as a therapeutic agent due to their effective treatment of ALL [12,15]. However, allergic reaction and significant side effects caused by these enzyme preparations have led us to find out novel sources of this enzyme from plants. Our group has succeeded in isolating L-asparaginase with high specific activity (850 IU/mg protein) and with no glutaminase activity from *Phaseolus vulgaris* seeds instead of microbial sources [18].

**Figure 1:**
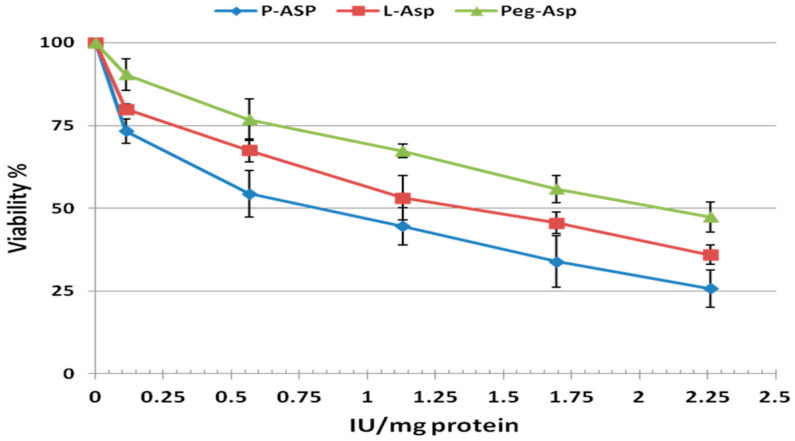
WST-8 assay for cell viability of Jurkat cells after treatment with adjusted doses of enzyme preparations according to their specific activity.

**Figure 2:**
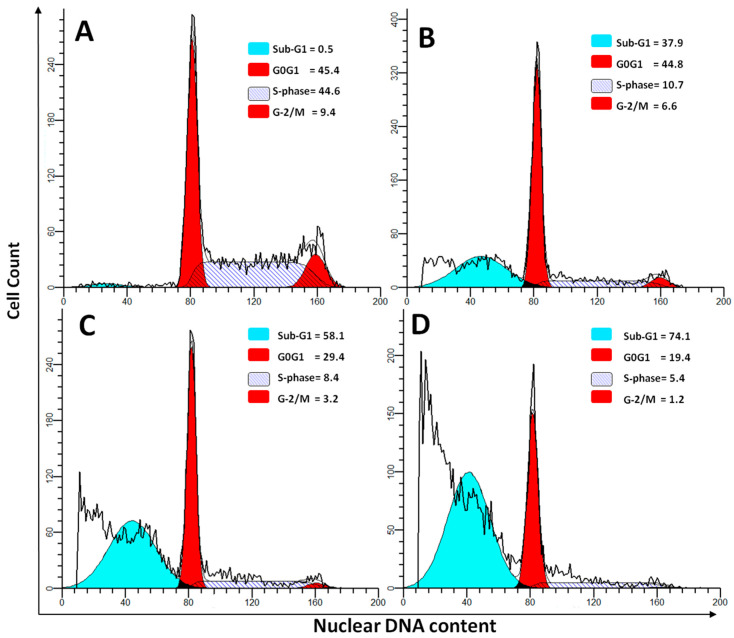
Representative DNA-flow cytometry histograms of Jurkat cells demonstrates cell cycle compartments after staining with DNA-specific fluorochrome Propidium iodide. (**A**) Control untreated cells showing high S-phase percent; (**B**) Jurkat cells treated with 0.113 IU/mg P-Asp; (**C**) Cells treated with P-Asp at 0.56 IU/mg protein; (**D**) Cells treated with P-Asp at a concentration of 2.25 IU/mg protein.

In the present study we compared the anti-cancer properties between P-Asp and other commercially available enzyme preparations L-Asp and PEG-Asp. Figure 1 represents a plot of percentage of cell viability and various concentrations of each enzyme were used to calculate the IC_50_, which represents the concentration possessing 50% anti-proliferation. P-Asp showed an enhanced anti-proliferative effect and lower IC_50_ (0.75 IU/mg) compared with IC_50_ of 1.3 and 2 IU/mg protein for L-Asp and Peg-Asp, respectively. This enhanced effect of anti-proliferative activity was also found in the cell cycle assay using DNA-flow cytometry (Figure 2). Administration of enzyme preparations leads to variable effects on cell cycle phases after staining with DNA specific dye Propidium iodide.

Table 1 demonstrates the difference in variance between these enzymes and shows that P-Asp induced significant reduction in S-phase compared with untreated control cells and with cells treated with L-Asp or Peg-Asp (*p* < 0.001, *p* < 0.05, *p* < 0.01, respectively). In addition, treatment with P-Asp induced a significantly higher number of apoptosis in a dose dependent manner compared with L-Asp and PEG-Asp (both at *p* < 0.001). This increased rate of apoptosis in cells treated with P-Asp may be attributed to increased activity in depleting asparagine or in lowering its synthesis as previously suggested by Ueno and Ohtawa [10].

**Figure 3:**
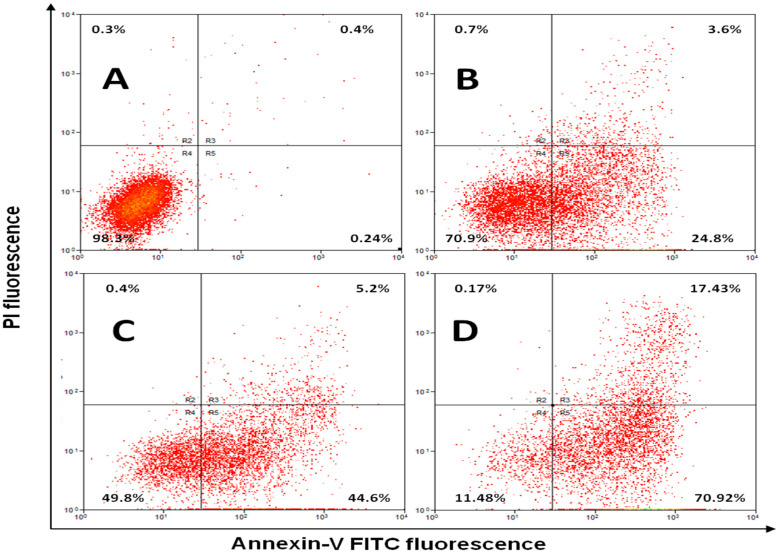
Annexin V-FITC and PI (propidium iodide) staining to evaluate apoptosis of Jurkat cells induced by various doses of P-Asp. (**A**) Untreated control cells; (**B**) Jurkat cells treated with 0.113 IU/mg P-Asp; (**C**) Cells treated with P-Asp at 0.56 IU/mg protein; (**D**) Cells treated with P-Asp at concentration of 2.25 IU/mg protein. In each panel the lower left quadrant shows live cells, which are negative for both PI and annexin V-FITC, the upper left quadrant shows only PI positive cells, which are necrotic. The lower right quadrant shows annexin positive cells (early apoptotic) and the upper right quadrants shows annexin and PI positive cells (late apoptosis cells).

To confirm these DNA cell cycle data, we analyzed the expression of the apoptotic marker annexin-V using flow cytometry. Annexin V is a 35–36 kDa Ca^2+^ dependent phospholipid-binding protein that a has high affinity for the phospholipid phosphatidylserine (PS) contained in the cytoplasmic surface of the cell membrane. Expression of PS at the cell surface occurred when cells undergo apoptosis [25]. Analysis of Annexin V expression was suggested to detect one of the earliest events in apoptosis—the externalization of PS—in living cells [26]. Figure 3 shows that treatment of variable doses of P-Asp induced a significant increase in early apoptosis in a dose dependent manner, and yet no necrosis occurs.

Table 2 demonstrates that at IC_50_ specific for each enzyme, P-Asp induced significantly higher early apoptosis compared with L-Asp and PEG-Asp (both at *p* < 0.05). Induction of apoptosis by L-asparaginase has been suggested previously in leukemic lymphoblasts as a result of depletion of the exogenous sources of asparagine, given the fact that most leukemic cells lack asparagine synthetase activity, the resulting lacks of asparagine leads to apoptosis and cell deaths [10,27].

**Figure 4:**
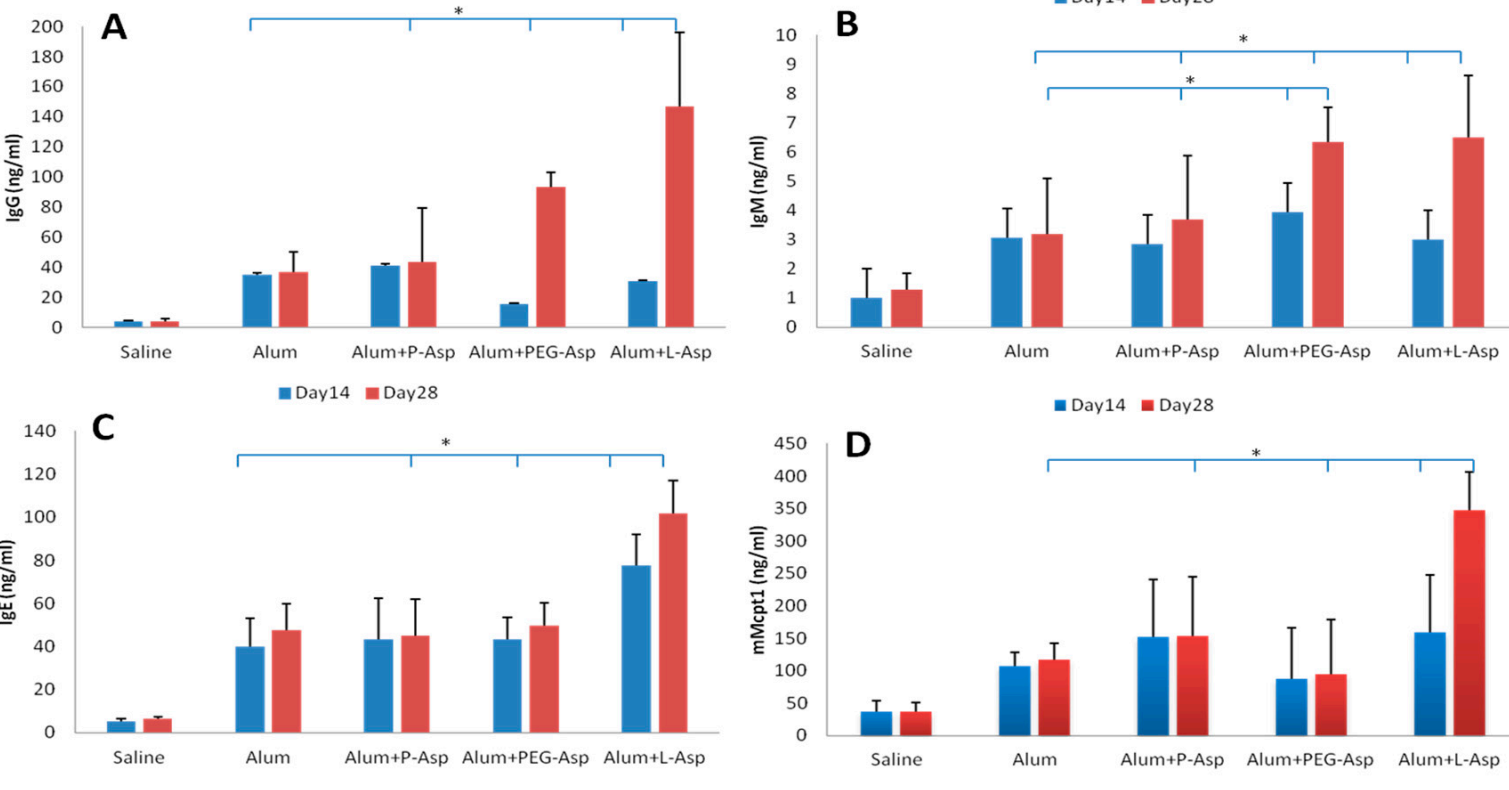
Analysis of immunogenic responses in Balb/c mice treated with Alum plus 50 and 100 IU/mg protein of either P-ASP, L-Asp or PEG-Asp at day 14 and day 28 respectively post initial sensitization with Alum. (**A**) Presents serum IgG levels; (**B**) Presents serum IgM levels; (**C**) Presents IgE in serum and (**D**) presents levels of mMCP-1 in serum. Data represent the average and error bars represents standard deviation. * Statistically significant at *p*  <  0.05 (One-way ANOVA with Dunnett’s test).

Patients treated with L-asparaginase prepared from bacterial origin develop serious side effects due to the development of antibodies against foreign bacterial proteins, especially upon re-exposure to asparaginase after an initial exposure [28,29]. Additionally, antibodies, instead of leading to clinical hypersensitivity, might cause rapid decline in asparaginase activity and lower efficiency to deplete asparagine. These events are referred to as “silent inactivation” that occur approximately in 30% of the patients [30]. To examine the immunogenicity of P-Asp, we performed an in-vivo experiment in five groups of mice that were injected with vehicle, Alum adjuvant, or 50 IU/mg of P-Asp, L-Asp, and PEG-Asp at day 14 and 100 IU/mg of the different enzymes at day 24 as described in methods. Figure 4 demonstrates that no significant changes in serum levels of immunoglobulins IgG and IgM were detected in mice treated with P-Asp. On the other hand, a significant increase in IgG titer in-group treated with L-Asp was detected at day 28 compared with other treatment groups. 

Liu et al. [31] reported that the increase in serum anti-asparaginase IgG levels occurs before the onset of allergic reactions and can neutralize asparaginase activity in serum of ALL patients. In addition, allergic reactions attributed to L-asparaginase treatment have involved cell-associated antigen-specific IgE antibodies and are mediated by the release of histamine from activated mast cells [32]. In the present study, IgG, IgM, IgE and mMCP-1 in mouse serum were found to be significantly increased following exposure to L-ASP. These findings are in agreement with the work of Fernandez and co-investigators [23]. On the other hand, the mice group treated with P-Asp showed no changes in IgE and in mouse mast cells protease-1 concentrations compared with mice treated with adjuvant, suggesting that no allergic reaction was developed. In the same manner, no increases in levels of either IgE or MCP-1 occurred in the group treated with PEG-Asp. These observations are in line with previous studies reporting that allergic reaction are less observed on treatment with PEG-Asp [16].

## 4. Conclusions

Our study demonstrates that L-asparaginase which isolated from *Phaseolus vulgaris* (P-Asp) has enhanced anti-proliferative and exhibits a higher degree of early apoptosis, compared to necrosis or late apoptosis of the Jurkat leukemic cell line. In addition, in-vivo experiments show that mice treated with P-Asp had less distinct immune responses as indicated by lower serum concentrations of IgG, IgE, IgM and mMCP-1 compared with other treated groups. Hence, P-Asp could be considered as a promising candidate for use in treatment of ALL.

## Figures and Tables

**Table 1:** DNA cell cycle phases of Jurkat cells treated with P-Asp, L-Asp and PEG-Asp at a concentration of 1 × IC_50_.

Phase	Untreated	P-Asp	L-Asp	PEG-Asp
Sub-G1	0.76 ± 0.04	58.43 ± 6.42 ^a,b,c^	33.75 ± 6.45 ^a^	29.58 ± 2.74 ^a^
G0G1	49.57 ± 7.35	29.43 ± 2.54 ^a,b,c^	43.54 ± 7.45	42.4 ± 6.72
S-Phase	41.51 ± 9.47	8.47 ± 1.1 ^a,b,c^	16.25 ± 3.75 ^a^	19.3 ± 2.01 ^a^
G2/M	8.14 ± 1.21	3.62 ± 0.52 ^a,b,c^	6.42 ± 2.02	5.65 ± 2.51

Values represent the average ± standard deviation of three different experiments. ^a^ significant compared with untreated control cells; ^b^ significant compared with cells treated with L-Asp; ^c^ significant compared with cells treated with PEG-Asp. Statistical significance was set at the 0.05 probability level.

**Table 2:** Annexin V apoptosis detection in Jurkat cells treated with P-Asp, L-Asp and PEG-Asp at a concentration of 1 × IC_50_.

Phase	Untreated	P-Asp	L-Asp	PEG-Asp
Early Apoptosis	0.65 ± 0.11	49.3 ± 11.08 ^a,b.c^	31.42 ± 11.57 ^a^	27.36 ± 8.35 ^a^
Late Apoptosis	0.58 ± 0.13	5.28 ± 1.54 ^a,b.c^	17.82 ± 9.31	13.75 ± 4.39
Necrosis	0.49 ± 0.09	1.03 ± 0.31 ^a,b.c^	5.71 ± 3.75 ^a^	9.14 ± 1.91 ^a^
Live cells	98.14 ± 1.87	44.32 ± 9.28 ^a^	44.86 ± 12.37	49.41 ± 11.9

Values represent average ± standard deviation of three different experiments. ^a^ Significant compared with untreated control cells; ^b^ significant compared with cells treated with L-Asp; ^c^ significant compared with cells treated with PEG-Asp. Statistical significance was set at the 0.05 probability level.
